# Hexapeptide-Liposome Nanosystem for the Delivery of Endosomal pH Modulator to Treat Acute Lung Injury

**DOI:** 10.3390/jfb16120450

**Published:** 2025-12-01

**Authors:** Yuting Ji, Qian Wang, Rujing Lin, Mimi Pang, Liya Sun, Jiameng Gong, Huiqiang Ma, Shan-Yu Fung, Hong Yang

**Affiliations:** 1 Department of Pharmacology and Tianjin Key Laboratory of Inflammatory Biology, The Province and Ministry Co-Sponsored Collaborative Innovation Center for Medical Epigenetics, School of Basic Medical Sciences, Tianjin Medical University, No. 22 Qixiangtai Road, Heping District, Tianjin 300070, China; 2 State Key Laboratory of Experimental Hematology, Key Laboratory of Immune Microenvironment and Disease (Ministry of Education), Department of Immunology, School of Basic Medical Sciences, Tianjin Medical University, No. 22 Qixiangtai Road, Heping District, Tianjin 300070, China; 3 School of Biomedical Engineering, Tianjin Medical University, Tianjin 300070, China

**Keywords:** peptide modification, lipid-based nanocarriers, hydroxychloroquine, lung inflammation, macrophage, Toll-like receptor

## Abstract

The overactivation of endosomal Toll-like receptor (TLR) in macrophages plays an important role in the pathogenesis of acute lung injury (ALI). There is currently still a lack of nano-formulated and macrophage-targeted endosomal TLR inhibitors that have been approved for clinical uses. We previously discovered that the elevation of endosomal pH using nanodevices provides a promising strategy to specifically inhibit endosomal TLRs in macrophages. The weakly basic drug hydroxychloroquine (HCQ) has been reported for its capability to accumulate in endolysosomes and modulate the acidity in these compartments. To enhance its macrophage-targeting ability and the therapeutic efficacy in vivo, herein we formulated HCQ into a nanoform using liposomes, named HCQ-L. We found that HCQ-L was less cytotoxic and more effective in inhibiting endosomal TLRs (including TLR3, TLR4, TLR 7/8) than the molecular HCQ. Subsequently, a hexapeptide, Pep12, was inserted onto the surface of HCQ-L to form HCQ-L-P12. Interestingly, Pep12 modification significantly improved the stability of liposomes in aqueous solution for at least 2 years; while having enhanced inhibitory effects on TLR7/8 signaling, HCQ-L-P12 displayed similar effects on inhibiting the TLR4 pathway and down-stream pro-inflammatory cytokine production when compared with HCQ-L. Furthermore, both HCQ nanoformulations potently elevated the endosomal pH. In vivo evaluation showed that HCQ-L-P12 and HCQ-L (but not molecular HCQ) were able to alleviate lung inflammation and injuries by decreasing inflammatory cell infiltration upon intratracheal instillation in a lipopolysaccharide (LPS)-induced acute lung injury (ALI) mouse model. This research provides a new strategy to fabricate lipid-based nanocarriers for targeted delivery of endosomal pH modulators to treat ALI and other acute and chronic inflammatory disorders.

## 1. Introduction

Acute lung injury (ALI) and its more severe form, acute respiratory distress syndrome (ARDS), represent life-threatening clinical conditions characterized by uncontrolled pulmonary inflammation, alveolar–capillary barrier damage, and progressive respiratory failure [1,2]. Despite great advances in supportive care, the mortality rate of ALI/ARDS still remains high (35~45%), which represents as a critical unmet need for effective pharmacological interventions that can efficiently target the underlying pathogenic mechanism for uncontrolled inflammation [3,4].

A pivotal driver of the uncontrolled inflammation in ALI is the aberrant activation of Toll-like receptor (TLR) signaling pathways in macrophages, particularly those present within endosomal compartments, such as TLR3, TLR4, TLR7/8, and TLR9 [5,6]. Among these, TLR4 recognizes endotoxins released from bacteria, and TLR3, TLR7/8, and TLR9 sense endogenous nucleic acids released from damaged cells, perpetuating a vicious cycle of pro-inflammatory cytokine production (e.g., TNF-α, IL-6) and neutrophil infiltration. Hydroxychloroquine (HCQ), a well-known antimalarial and immunomodulatory drug, functions as a weak base that accumulates in acidic organelles, including endosomes and lysosomes, and effectively modulates the endosomal pH, thereby disrupting the necessary acidic environment for endotoxin and nucleic acid ligand recognition and signal transduction during endosomal TLR activation [7]. However, the clinical efficacy of HCQ in ALI has been largely limited by its poor pharmacokinetics, non-specific tissue distribution, and dose-related systemic toxicity, necessitating a targeted delivery strategy to enhance its therapeutic index [8,9,10].

Nanoparticle-based drug delivery systems, particularly liposomes, offer a compelling solution to the challenges of HCQ applications in ALI. Their biodegradable and biocompatible nature, coupled with the ability to encapsulate both hydrophilic and hydrophobic agents, makes them ideal carriers for improving drug pharmacokinetics and pharmacodynamics [11]. For inflammatory pulmonary diseases, appropriately designed liposomes can passively target the inflamed pulmonary vasculature [12,13]. Nevertheless, conventional liposomes suffer from inherent instability in aqueous environments, often leading to premature drug leakage, aggregation, and fusion during storage and in vivo applications. This instability severely compromises the dosing accuracy and therapeutic efficacy, representing a major bottleneck in their clinical translation [14].

To overcome this limitation, various surface modification strategies have been used. Among them, peptide-based modifications have gained attention for their ability to confer specific functionalities—from enhancing stability to enabling active targeting [15,16,17]. Previously, we identified a novel hexapeptide with the amino acid sequence of CLPFFD (Pep12), which can stabilize gold nanoparticles in physiological conditions, enhance nanoparticle cellular uptake in macrophages, and enable the modulating capability on TLR signaling upon being coated onto gold nanoparticles, while the free form has no such bioactivity [18,19,20]. Using the advantages of Pep12, in this study, we aimed to introduce this hexapeptide onto liposomes to improve the stability and therapeutic efficacy of HCQ-encapsulated liposomes.

Herein, we first constructed HCQ-loaded liposomes (as HCQ-L) and evaluated their capability of inhibiting endosomal TLR signaling in macrophages in comparison with the free drug. We further modified this nanosystem with Pep12, generating HCQ-L-P12, and demonstrated that this modification significantly improved the long-term stability while preserving the inhibitory function of HCQ-L on endosomal TLR pathways and pro-inflammatory cytokine production through modulating endosomal pH. Finally, in a murine model of LPS-induced acute lung injury (ALI), we confirmed that HCQ-L-P12 effectively attenuated pulmonary inflammation comparable to HCQ-L. Such hexapeptide-modified liposomal nanocarriers represent a robust and translatable nanotherapeutic strategy for the targeted delivery of pharmaceutical compounds to treat ALI/ARDS and other inflammatory disorders.

## 2. Experiments and Methods

### 2.1. Materials

Soya lecithin and cholesterol were obtained from Shanghai Taiwei Pharmaceutical Co., Ltd. (Shanghai, China) and Sangon Biotech (Beijing, China), respectively. DSPE-PEG_2000_-MAL was obtained from AVT (Shanghai, China). Hydroxychloroquine sulfate was purchased from Tixiai Chemical Industry Development Co., LTD (Shanghai, China). The hexapeptide Pep12 (CLPFFD) was synthesized by Nanjing Jietai Biological Co., Ltd. (Nanjing, China). Ammonium sulfate was purchased from Aladdin Biochemical Technology Co., LTD (Shanghai, China). Chloroquine (CQ) was from Sigma (Sant-Louis, MO, USA). The phosphate-buffered saline (PBS), RPMI 1640 culture medium, and fetal bovine serum (FBS) were acquired from Biological Industries (Kibbutz Beit Haemek, Israel), whereas the sodium pyruvate and L-glutamine were purchased from Gibco (Grand Island, NY, USA). The human THP-1 monocytic cell line was obtained from ATCC (Rockefeller, MD, USA). The THP-1 XBlue and THP-1 ISG reporter cell lines, QUANTI-Blue™ solution, Zeocin, phorbol 12-myristate 13-acetate (PMA), Pam3CSK4, Poly I:C (high molecular weight), lipopolysaccharide (LPS), and resiquimod (R848) were all obtained from InvivoGen (San Diego, CA, USA). The MTS assay was from Promega (Madison, WI, USA). The primary antibodies against IκBα (9242S), phosphorylated p65 (p-p65, 3033S), β-actin (8457S), and phosphorylated IRF3 (p-IRF3, 4947S), as well as the HRP-linked anti-rabbit antibody (7074S), were all acquired from Cell Signaling Technology (Boston, MA, USA). The RIPA buffer for cell lysis, Coomassie Plus (Pierce) for protein concentration measurement, Halt protease and phosphatase inhibitor cocktail, fluoresceine-dextran (10,000 MW), and pHrodo red-dextran were purchased from Thermo Fisher Scientific (Waltham, MA, USA). The Human ELISA kits of IL-6, MCP-1, and TNF-α were obtained from eBioscience (San Diego, CA, USA). Liu’s stain and red blood cell (RBC) lysis buffer were obtained from Solarbio Science & Technology (Beijing, China).

### 2.2. Preparation and Characterization of HCQ-L and HCQ-L-P12

HCQ-L was synthesized by the classic thin-film hydration method. Cholesterol (60 mg) and soya lecithin (180 mg) were dissolved in chloroform first, and then the solvent was completely evaporated to form the thin film. It was rehydrated in an ammonium sulfate solution (15 mL, 300 mM) at 37 °C for 30 min, which was sonicated by a bath ultrasonicator (Scientz, Ningbo, China) at 150 W for 30 min; the suspensions were then dialyzed for 24 h at room temperature to obtain liposomes. The unloaded liposomes were incubated with 5.5 mg HCQ for 30 min at 40 °C to allow HCQ to actively enter into liposomes through the ammonium sulfate gradient method to obtain HCQ-loaded liposomes (HCQ-Ls). For HCQ-L-P12 construction, M-P12 was first prepared. Briefly, chloroform (2 mL) was used to dissolve the PEGylated lipid-based monomers DSPE-PEG_2000_-Mal (10 mg). The above solution was dried at low pressure at 45 °C to obtain a thin film. PBS (3 mL) was then used to rehydrate the film at 55 °C, followed by bath ultrasonication (Scientz, Ningbo, China) at room temperature with a power of 120 W for 5 min to obtain the nanomicelles. The Pep12 (CLPFFD) solution (2 mL, 2 mg/mL in PBS) was added to the nanomicelles at a molar ratio of 1.5:1 (Pep12:DSPE-PEG_2000_-MAL). With gentle stirring at room temperature in the dark for 24 h, the sulfhydryl group of Pep12 was conjugated to the maleimide group of DSPE-PEG_2000_-MAL by the Michael addition reaction. The resulting mixtures were dialyzed against PBS (pH 7.4) for 24 h to remove the unconjugated Pep12 to obtain M-P12. HCQ-Ls were incubated with M-P12 for 2 h at 60 °C to allow for the insertion of M-P12 monomer onto the liposomes to obtain HCQ-L-P12. All liposomes were sonicated by a probe ultrasonicator (Scientz, Ningbo, China) at 100 W for 6 min (ultrasonicate for 1 s and then stop for 2 s), sterilized by filtration (0.22 mm, Millipore, Billerica, MA, USA), centrifugated at 14,000 rpm for 2 h, and stored at 4 °C prior to use.

The physicochemical properties of HCQ-L and HCQ-L-12 were characterized using the following techniques. First, the nanoscale morphology was visualized using a transmission electron microscope (TEM) (HT7700, Hitachi, Tokyo, Japan) with an accelerating voltage of 80 kV. Next, the hydrodynamic diameter and zeta potential were acquired using a Zetasizer instrument (Nano ZS, Malvern, Worcestershire, UK). For DLS analysis, the sample was measured with a monochromatic laser beam (632.8 nm), and the scattered light was detected at 173° from the incident light. The hydrodynamic diameter distribution was analyzed using the setting of “General Purpose” (multimodal mode) to generate the envelope curves.

### 2.3. Encapsulation Efficiency and Drug Loading Efficiency of HCQ

HCQ-L and HCQ-L-P12 were centrifugated (14,000 rpm, 4 °C, 1 h) twice, and the supernatants were collected for the measurement of absorption at 343 nm on a microplate reader (TECAN, Mannedorf, Zurich, Switzerland). The unencapsulated HCQ content was calculated according to the standard curve, recorded as W1. The formulas used to calculate encapsulation efficiency (EE) and drug loading (DL) efficiency are as follows:

EE (%) = (W2 − W1)/W2 × 100%

DL (%) = (W2 − W1)/W3 × 100%

where W2 is the total mass of HCQ; W3 is the mass of liposomes.

### 2.4. Drug Release Profile

Liposomes (1 mL) were dialyzed in 20 mL of PBS and under continuous shaking (100 rpm) at 37 °C. A fixed volume (0.5 mL) of PBS was taken at different time points of 0, 0.5, 1, 2, 4, 8, 12, 16, and 24 h to determine the presence of HCQ in PBS by absorption measurement at 343 nm on a microplate reader (TECAN, Mannedorf, Zurich, Switzerland). The concentration of HCQ in PBS at different time points was estimated using a standard curve to obtain the release profile.

### 2.5. Culture of THP-1 Cell-Derived Macrophages

THP-1 cells were cultured in the complete culture medium made of the RPMI-1640 medium with 10% FBS, sodium pyruvate (1 mM), and L-glutamine (2 mM) at 37 °C, 5% CO_2_. The THP-1-ISG and THP-1-XBlue reporter cells were maintained in the complete culture medium with the addition of Zeocin (200 and 100 μg/mL, respectively) every other passage to ensure the selection pressure. Cells were seeded into a 96-well or 24-well plate and differentiated into macrophages with the addition of PMA (50 ng/mL) for 24 h. These cells were further washed with PBS twice and rested for 48 h before further use.

### 2.6. Reporter Cell Assay for the NF-kB/AP-1 and IRF Activation Analysis

Reporter cells (1 × 10^5^ cells/well) were seeded in a 96-well plate and differentiated into macrophages. They were treated with different TLR ligands in the presence or absence of HCQ, HCQ-L, or HCQ-L-P12 for 24 h; 20 μL of the culture medium was collected to mix with 180 μL of QUANTI-Blue solution in a 96-well plate, which was incubated at 37 °C until the color changed. A microplate reader (TECAN, Mannedorf, Zurich, Switzerland) was used to measure the absorption at 655 nm. The color change from pink to dark blue indicated the activation of NF-κB/AP-1 or IRF in comparison with the untreated control.

### 2.7. Immunoblotting Analysis

THP-1 cells (5 × 10^5^ cells/well) were seeded into a 24-well plate and differentiated into macrophages. They were stimulated with LPS (10 ng/mL) with or without HCQ-L or HCQ-L-P12 treatment for various time periods. The ice-cold RIPA buffer consisting of the Halt protease and phosphatase inhibitor cocktail was used to lyse the cells. For each sample, the total protein concentration was quantified using the Bradford assay and adjusted equally. A 10% SDS-PAGE was used to separate the proteins, which were then transferred to a PVDF membrane. The membranes were blocked with the washing buffer (TBS buffer with 0.1% Tween 20, TBST) containing 5% BSA for 1 h at room temperature; they were then blotted with primary antibodies of interest at 4 °C overnight. After washing with TBST thoroughly, the membranes were blotted with HRP-labeled antibody for 1 h at room temperature. Finally, the chemiluminescence method (ECL, Millipore, Billerica, MA, USA) was applied to probe the protein bands using a ChemiDoc MP imaging system (Bio-Rad, Hercules, CA, USA). The levels of the protein bands were quantified by the densitometry analysis using ImageJ software 1.52a (NIH, Bethesda, MD, USA).

### 2.8. Cytokine Analysis

THP-1 cells were seeded (5 × 10^5^ cells/well) and differentiated into macrophages in a 24-well plate. Cells were treated with LPS with/without HCQ-L or HCQ-L-P12 for 24 h; the culture medium of each well was harvested and centrifuged (14,000 rpm, 4 °C, 30 min) to collect the supernatants. The levels of IL-6, TNF-α, and MCP-1 in these supernatants were quantified by ELISA using commercial kits following the manufacturer’s instructions.

### 2.9. Endosomal pH Assessment

THP-1 cells (2.4 × 10^5^ cells) were cultured in a 20 mm glass bottom dish (NEST, Wuxi, China) for confocal imaging and differentiated into macrophages. The cells were treated with pHrodo red-dextran (10 μg/mL) and fluorescein-dextran (20 μg/mL) for 1 h before the addition of HCQ-L, HCQ-L-P12 (50 μM), or CQ (30 μM) overnight. After washing with PBS 3 times, cells were imaged on a LSM900 confocal microscope (Zeiss, Wetzlar, Hessen, Germany). The fluorescence intensities of fluorescein (ex: 488 nm; em: 525 nm) and pHrodo red (ex: 565 nm; em: 585 nm) in each cell were quantified via Image J software. For each condition, the intensity ratios of fluorescein to pHrodo red were calculated from at least 40 cells of two independent experiments.

### 2.10. LPS-Induced ALI Mouse Model

The C57BL/6 mice (male, 6–8 weeks) were purchased from SPF Biotechnology Co., Ltd. (Beijing, China). The mouse studies were carried out following the guidelines of the Institutional Animal Care and Use Committee of Tianjin Medical University (TMUaMEC2020004). The mice were housed 5 mice/cage and adaptively fed for 1–2 weeks prior to experiments. The mice were anesthetized with 1% sodium pentobarbital for all operation procedures. Mice were randomly put into five different groups: PBS+PBS, PBS+LPS, HCQ+LPS, HCQ-L+LPS, and HCQ-L-P12+LPS. Under the anesthetized condition, the mouse trachea was exposed by a median neck incision first, and then HCQ/HCQ-L/HCQ-L-P12 solution (containing 235.8 μM HCQ, 50 mL/mouse) or an equal volume of PBS was injected into the trachea 1 h before the intratracheal administration of LPS (10 mg/kg) (Sigma-Aldrich, Sant-Louis, MO, USA). Twenty-four hours after LPS challenge, the bronchoalveolar lavage fluid (BALF) and lung tissues were collected to examine the lung inflammation and injury. Data from all tested mice were analyzed with no exclusions. To minimize the number of mice used, only three mice were assigned to the control group due to the relatively low variance.

### 2.11. BALF Collection and Differential Cell Counting

The collected BALF solution of mice was first centrifuged at 10,000 rpm for 30 s at 4 °C. The cell pellets were then treated with the red blood cell lysis buffer and resuspended in PBS for cell counting analysis. The total number of cells was counted on a hemocytometer. Subsequently, cells from the suspension were deposited onto a glass slide using a cytospin (Thermo Fisher Scientific, Waltham, MA, USA) and stained with Liu’s stain. Differential cell counts were performed on the stained slides under an upright microscope (Nikon, Tokyo, Japan) at 400× magnification. A minimum 300 cells were enumerated for each sample.

### 2.12. Lung Histology and Injury Score

At the end of the ALI mouse model, the right lower lung lobe was collected and fixed in 4% paraformaldehyde, followed by dehydration, paraffin embedding, and sectioning. These sections were stained with hematoxylin and eosin (H&E), and their images were acquired under an optical microscope (Nikon, Tokyo, Japan). For each sample, more than 20 images at 400× magnification were blindly assessed by at least two independent researchers to obtain the lung injury scores based on five histopathological features: alveolar neutrophils, interstitial neutrophils, hyaline membranes, proteinaceous debris, and alveolar septal thickening. Each indicator was rated as 0, 1, or 2 points based on the severity of the criteria. These five independent variables were then weighted according to their correlations with ALI. The score was further normalized by the number of visual fields which represents the overall score of lung tissue injury for each mouse. The total score of lung injury and the scores of each individual feature of each mouse were graphed separately for statistical analysis.

### 2.13. Statistical Analysis

The statistical analysis was performed using GraphPad Prism 9.0.0 (121) (last updated on 22 October 2020, GraphPad Software LLC). The mean ± standard error of mean (SEM) was used for all data presentation in this study. One-way ANOVA with Bonferroni’s post hoc test was applied for multiple comparison analysis. A *p*-value less than 0.05 was defined as the threshold for statistical significance.

## 3. Results

### 3.1. Fabrication of the HCQ-Encapsulated Liposomal Nanosystem and Evaluation of Its Inhibitory Activity on TLR4 Signaling Pathway

**Figure 1:**
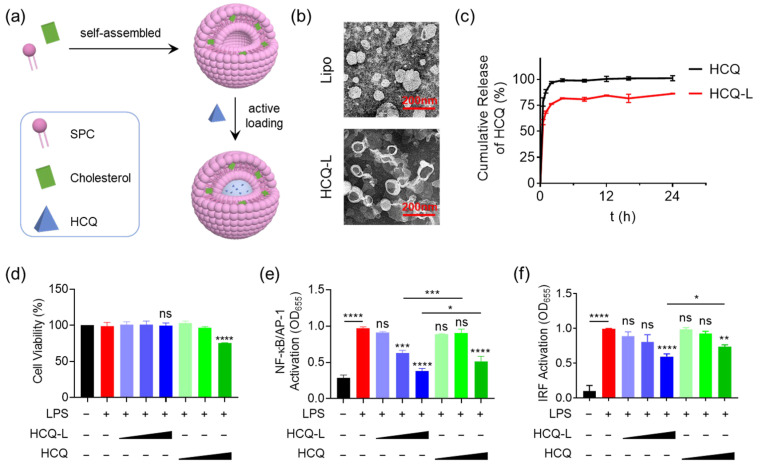
Fabrication of the HCQ-encapsulated liposomes (HCQ-L) and their inhibitory activity on TLR4 signaling. (**a**) A schematic diagram showing the fabrication process of HCQ-L; HCQ was actively loaded into liposomes through the ammonium sulfate gradient method; SPC: sphingosylphosphorylcholine. (**b**) The TEM images showing the size and morphology of the empty liposomes (Lipo) and HCQ-L; scale bar = 200 nm. (**c**) The cumulative drug release profile of free HCQ and HCQ-L. *N* = 3. (**d**) The effects of HCQ and HCQ-L on cell viability with different HCQ concentrations under LPS stimulation; *N* = 3; ns: not significant, **** *p* < 0.0001 vs. unstimulated group. (**e**,**f**) The concentration-dependent effects of HCQ and HCQ-L on the activation of NF-κB/AP-1 (**e**) and IRF (**f**) in the THP-1 reporter cell-derived macrophages upon LPS stimulation. LPS = 10 ng/mL, HCQ/HCQ-L = 1, 10, 50 μM (HCQ concentration); *N* = 3 for NF-κB/AP-1 and *N* = 5 for IRF; ns: not significant, * *p* < 0.05, ** *p* < 0.01, *** *p* < 0.001, **** *p* < 0.0001 vs. LPS group unless otherwise specified.

In order to reduce potential cytotoxicity of HCQ and enhance the targeting capability to macrophages, we constructed an HCQ-encapsulated liposomal nanosystem (HCQ-L) using the thin-film hydration method together with the classical ammonium sulfate gradient method (Figure 1a). HCQ molecules were loaded inside the water phase of the liposomes. The TEM images demonstrated that the empty liposomes (Lipo) and HCQ-L had a spherical shape and relatively uniform size distribution with average diameters of 77.0 ± 29.2 nm and 83.1 ± 30.5 nm, respectively (Figure 1b). The HCQ release profile of HCQ-L in comparison with the molecular HCQ was then evaluated. As shown in Figure 1c, about 85% of the drug released from HCQ-L at 24 h, whereas more than 95% of the free drug released from the dialysis bag within 2 h. This indicates that the encapsulated HCQ in liposomes had a slower release profile than the free HCQ.

Next, we evaluated the cytotoxicity of HCQ-L in comparison with the free HCQ on THP-1 cell-derived macrophages. As expected, HCQ-L did not exhibit any toxicity to the cells with various HCQ concentrations from 1 mM to 50 mM (Figure 1d). For the free HCQ, however, the cell viability decreased significantly (to ~80%) at the highest HCQ concentration of 50 μM. These results demonstrate that the liposomal nanosystem could reduce the toxicity of free HCQ at high concentrations.

In addition to reducing cytotoxicity, the liposomal nanosystem could also enhance the inhibitory activity of HCQ on TLR4 signaling. Using the reporter cell system, it was found that HCQ-L was effective at inhibiting the activation of the transcription factors NF-κB/AP-1 (Figure 1e) and IRF (Figure 1f) under LPS stimulation in the THP-1 reporter cell-derived macrophages. Such an inhibitory effect of HCQ-L was more potent than the free HCQ (Figure 1e,f). This indicates that the liposomal nanosystem could enhance the drug potency of HCQ.

### 3.2. HCQ-L Had a Broad Inhibitory Activity on the Endosomal TLR Pathways

**Figure 2:**
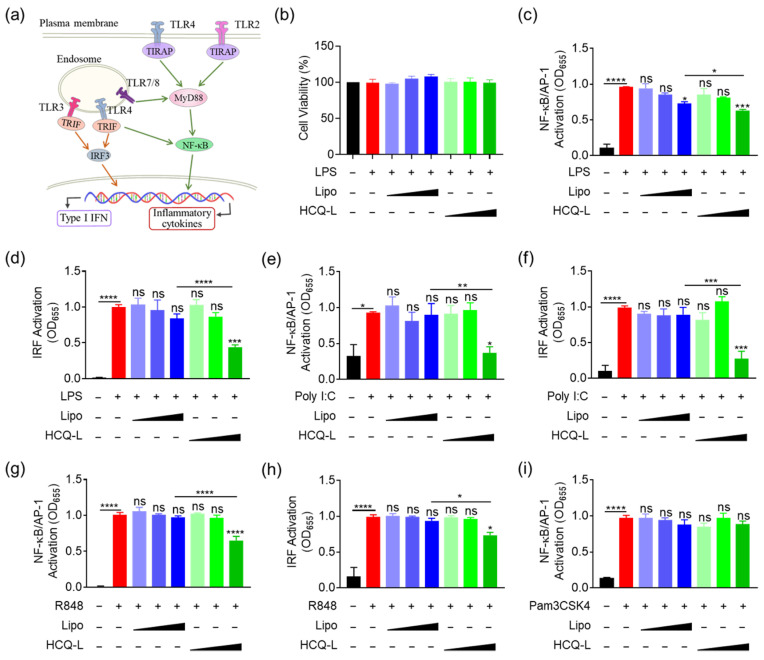
The preferential inhibitory activity of HCQ-L on endosomal TLR signaling pathways in THP-1 reporter cell-derived macrophages. (**a**) The schematic diagram of TLR2/3/4/7/8 signaling pathways. (**b**) The effects of HCQ-L and empty liposomes (Lipo) on the cell viability of the THP-1 reporter cell-derived macrophages. *N* = 3. (**c**–**h**) The effects of HCQ-L on the activation of NF-κB/AP-1 (**c**,**e**,**g**) and IRF (**d**,**f**,**h**) in the THP-1 reporter cell-derived macrophages upon LPS (for TLR4) (**c**,**d**), Poly I:C (for TLR3) (**e**,**f**), and R848 (for TLR7/8) (**g**,**h**) stimulation. (**i**) The effects of HCQ-L on the Pam3CSK4 (for TLR2)-induced activation. LPS = 10 ng/mL, Poly I:C = 50 μg/mL, R848 = 10 μg/mL, Pam3CSK4 = 10 ng/mL, L/HCQ-L = 1, 10, 50 μM; *N* = 3; ns: not significant, * *p* < 0.05, ** *p* < 0.01, *** *p* < 0.001, **** *p* < 0.0001 vs. TLR agonist groups unless otherwise specified.

Since HCQ is known for modulating endosomal pH, we aimed to examine the inhibitory activity of HCQ-L on endosomal TLR pathways. Among all TLRs, the TLR4 pathway includes two signaling cascades: (i) myeloid differentiation factor 88 (MyD88) dependent pathway to activate NF-κB/AP-1 and (ii) MyD88 independent pathway (via Toll/interleukin-1 receptor domain adapter-inducing interferon beta, TRIF) [21] to activate both NF-κB and IRF3 (Figure 2a). Using the THP-1 report cell-derived macrophages, we first demonstrated that HCQ-L and the empty liposomes did not cause any toxicity to the cells at the experimental concentrations (Figure 2b). It was found that HCQ-L inhibited both NF-κB/AP-1 (Figure 2c) and IRF (Figure 2d) activation in a concentration-dependent manner under LPS stimulation. Interestingly, the empty liposomes (Lipo) slightly inhibited LPS-induced NF-κB/AP-1 activation at the highest concentration (Figure 2c), owing to the intrinsic property of liposomes in scavenging and neutralizing extracellular LPS [22].

In addition to TLR4, we also evaluated the effects of HCQ-L on other endosomal TLR pathways such as TLR3 and TLR7/8 (Figure 2a). The stimulation on these TLRs can also lead to the activation of NF-κB and IRF. Upon different stimuli Poly I:C and R848 for TLR3 and TLR7/8 activation, respectively, it was found that HCQ-L (at a concentration of 50 μM) exhibited significant effects on suppressing NF-κB/AP-1 and IRF signals in both pathways (Figure 2e–h). However, HCQ-L had no effects on the Pam3CSK4-induced NF-κB/AP-1 activation in the cell surface TLR2 signaling pathway (Figure 2i). These results demonstrate that HCQ-L had a broad inhibitory spectrum preferentially for endosomal TLR pathways.

### 3.3. Fabrication and Characterization of Hexapeptide-Modified Liposomal Nanosystem

**Figure 3:**
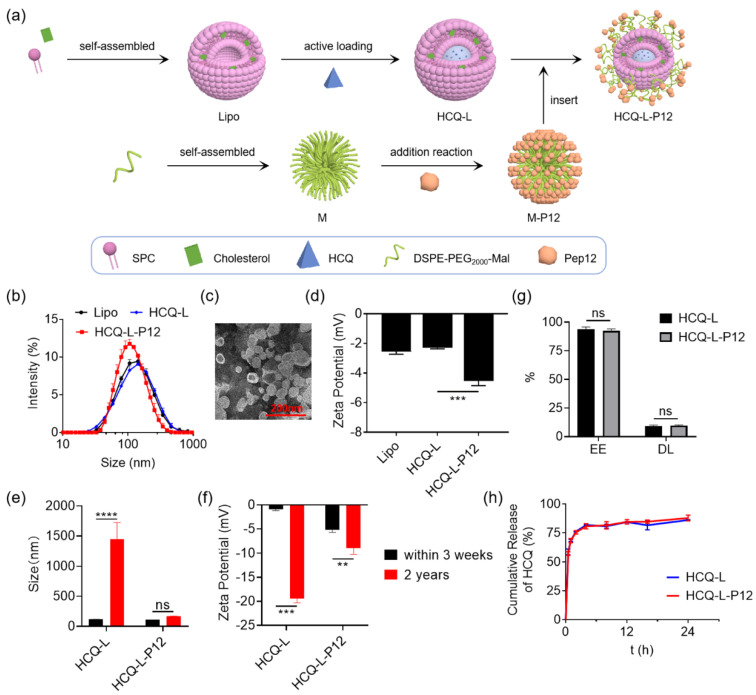
Fabrication and characterization of the hexapeptide-modified HCQ-L (HCQ-L-P12). (**a**) A schematic diagram showing the fabrication process of HCQ-L-P12. (**b**) The hydrodynamic diameter distributions of Lipo, HCQ-L, and HCQ-L-P12 by DLS analysis. (**c**) A representative TEM image showing the size and morphology of HCQ-L-P12; scale bar = 200 nm. (**d**) The Zeta potentials of Lipo, HCQ-L, and HCQ-L-P12. (**e**,**f**) The hydrodynamic sizes (**e**) and Zeta potentials (**f**) of HCQ-L-P12 in comparison with HCQ-L after short-term (within 3 weeks) and long-term (2 years) storage. (**g**) The encapsulation efficiency (EE) and drug loading efficiency (DL) of HCQ-L-P12 in comparison with the unmodified HCQ-L. (**h**) The cumulative drug release profiles of HCQ-L and HCQ-L-P12. *N* = 3, ns: not significant, ** *p* < 0.01, *** *p* < 0.001, **** *p* < 0.0001.

We previously developed a hexapeptide-modified lipid core nanomicelle M-P12 with robust activities on inhibiting multiple TLR pathways [23]. M-P12 was formed by the self-assembly of distearoyl-phosphatidylethanolamine-poly(ethylene glycol) (2000)-maleimide (DSPE-PEG_2000_-MAL) into nanomicelles with the hexapeptide Pep12 (CLPFFD) modification on the surface. The peptide modification was accomplished by conjugating Pep12 to DSPE-PEG_2000_-MAL through the Michael addition reaction between the thiol group of the cysteine (C) on the peptide and the maleimide group of the lipid. In this study, we aimed to take the advantages of Pep12 modification into our HCQ-L system to enhance the therapeutic effectiveness of HCQ-L. The Pep12 was introduced into HCQ-L by mixing M-P12 with HCQ-L to allow for the insertion of DSPE-PEG_2000_-Pep12 onto the surface of liposomes to obtain HCQ-L-P12 (Figure 3a).

The physicochemical properties of HCQ-L-P12 were first characterized in comparison with HCQ-L and empty liposomes (Lipo). The DLS analysis revealed that all Lipo, HCQ-L, and HCQ-L-P12 had similar size distributions with average hydrodynamic diameters of 120.1 ± 7.9 nm, 121.8 ± 10.7 nm, and 104.8 ± 10.8 nm, respectively (Figure 3b). The TEM image of HCQ-L-P12 also displayed a spherical morphology with an average size of 76.4 ± 24.9 nm (Figure 3c), similar to HCQ-L and Lipo (Figure 1b). The Zeta potential analysis showed that HCQ-L-P12 had a much more negative potential value (−4.5 ± 0.6 mV) than the unmodified HCQ-L (−2.3 ± 0.1 mV) and Lipo (−2.6 ± 0.3 mV) (Figure 3d), indicating the greater stability of HCQ-L-P12 [24]. We then examined the stability of HCQ-L-P12 in comparison with HCQ-L for short-term (within 3 weeks) and long-term (2 years) storage by analyzing their changes in particle sizes and Zeta potential values. It was found that the size of HCQ-L increased significantly while that of HCQ-L-P12 remained unchanged, even after long-term storage (Figure 3e). In addition, the Zeta potential value of HCQ-L became much more negative after long-term storage, indicating the formation of larger liposomes, whereas that of HCQ-L-P12 changed slightly (Figure 3f). Moreover, Pep12 modification had no effects on the encapsulation efficiency (EE, ~95%) and drug loading (DL, ~10%) efficiency of HCQ (Figure 3g); similarly, the drug release profile of HCQ-L-P12 was not affected by the Pep12 modification when compared with that of HCQ-L (Figure 3h).

### 3.4. The Inhibitory Activity of HCQ-L-P12 on Endosomal TLR Signaling, Cytokine Production, and Endosomal Acidification in Macrophages

**Figure 4:**
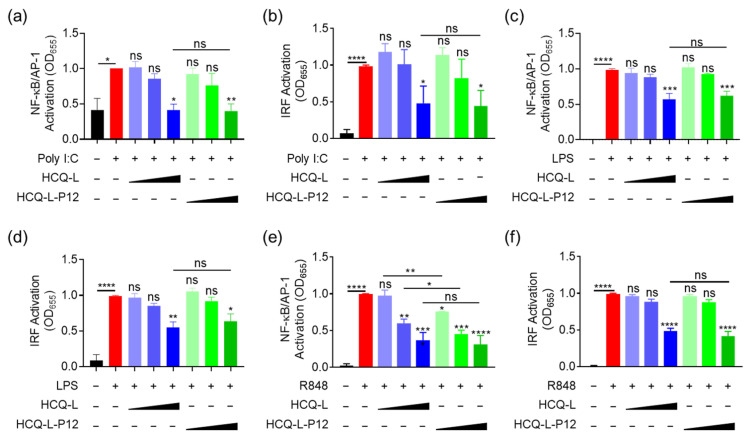
The inhibitory activity of HCQ-L-P12 on the endosomal TLR signaling pathways in THP-1 reporter cell-derived macrophages. (**a**–**f**) The effects of HCQ-L-P12 on the activation of NF-κB/AP-1 (**a**,**c**,**e**) and IRF (**b**,**d**,**f**) upon Poly I:C (50 μg/mL) (**a**,**b**), LPS (10 ng/mL) (**c**,**d**), and R848 (10 μg/mL) (**e**,**f**) stimulation for TLR3, TLR4, and TLR7/8 signaling pathway, respectively. HCQ-L/HCQ-L-P12 = 1, 10, 50 μM (HCQ concentration); N = 3, ns: not significant, * *p* < 0.05, ** *p* < 0.01, *** *p* < 0.001, **** *p* < 0.0001 vs. TLR agonists unless otherwise specified.

The inhibitory activity of HCQ-L-P12 on the endosomal TLR pathways were evaluated using the reporter cell system. Similar to HCQ-L, HCQ-L-P12 was able to down-regulate the activation of both NF-κB/AP-1 and IRF downstream of TLR3, TLR4, and TLR7/8 (Figure 4). At the high concentration (50 mM), HCQ-L-P12 showed similar potency in TLR3 and TLR4 inhibition to that of HCQ-L (Figure 4a–d). Interestingly, at the low concentrations (1 μM and 10 μM), HCQ-L-P12 appeared to have a stronger inhibitory effect on R848-induced NF-κB/AP-1 activation than HCQ-L (Figure 4e). These results demonstrate that HCQ-L-P12 also displayed a broad inhibitory activity on endosomal TLR pathways.

**Figure 5:**
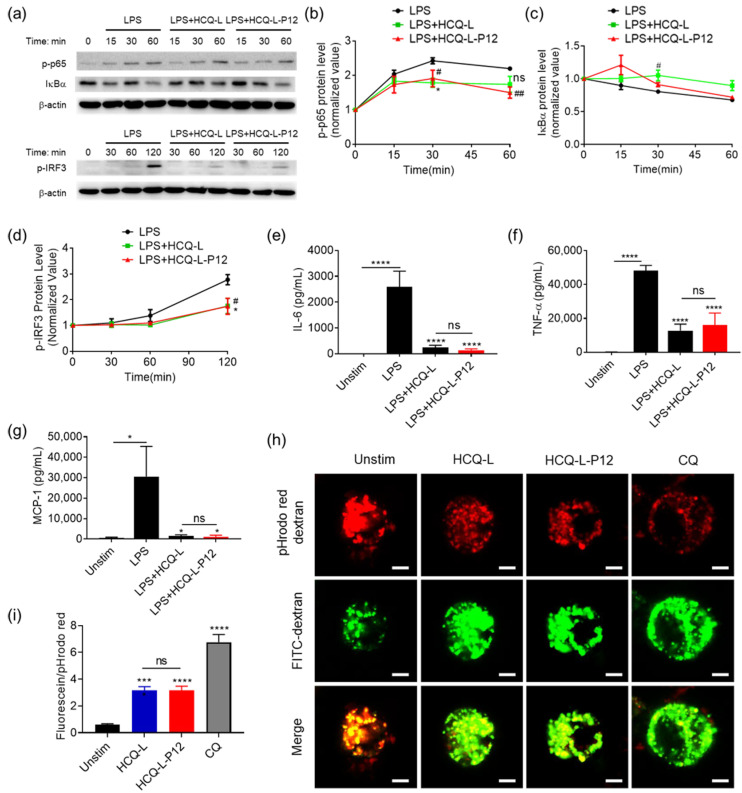
The inhibitory activity of HCQ-L and HCQ-L-P12 on NF-kB activation, pro-inflammatory cytokine production, and endosomal/lysosomal acidification. (**a**) The representative immunoblots showing the effects of HCQ-L and HCQ-L-P12 on the phosphorylation of p65 (p-p65) and IRF3 (p-IRF3) as well as the degradation of IκBα in the THP-1 cell-derived macrophages upon LPS stimulation over time (0–60 min); β-actin as the internal control. (**b**–**d**) The densitometry analysis of p-p65 (**b**), IκBα (**c**), and p-IRF3 (**d**) from the immunoblots in (**a**); *N* = 3; ns: not significant, #, * *p* < 0.05, ## *p* < 0.01 vs. LPS group. (**e**–**g**) The effects of HCQ-L and HCQ-L-P12 on the LPS-induced production of the pro-inflammatory cytokines IL-6 (**e**), TNF-α (**f**), and MCP-1 (**g**) in THP-1 cell-derived macrophages; *N* = 4 (for IL-6), 5 (for TNF-α), and 3 (for MCP-1); ns: not significant, * *p* < 0.05, **** *p* < 0.0001 vs. LPS group unless otherwise specified. (**h**) The representative confocal images showing the effects of HCQ-L and HCQ-L-P12 on the endosomal acidification probed by pHrodo red (red) (10 μg/mL) and fluorescein (green) (20 μg/mL) labeled dextran in THP-1 cell-derived macrophages overnight; chloroquine (CQ, 30 μM) was used as the positive control; scale bar = 5 μm. (**i**) The ratio of fluorescein/pHrodo red fluorescence intensities quantified from (**i**); *N* ≥ 40 cells from 2 independent experiments; ns: not significant, *** *p* < 0.001, **** *p* < 0.0001 vs. untreated control unless otherwise specified. LPS = 10 ng/mL, HCQ-L/HCQ-L-P12 = 50 μM (HCQ concentration).

The inhibitory activity on the TLR4 pathway was further assessed by immunoblotting over time. It was found that HCQ-L could significantly decrease the LPS-induced NF-κB p65 phosphorylation (p-p65) at 30 min, whereas HCQ-L-P12 reduced p-p65 at both 30 and 60 min (Figure 5a,b). The degradation of IκBα (as an indicator of NF-κB activation) was inhibited by HCQ-L at 30 min (Figure 5a,c). Furthermore, both HCQ-L and HCQ-L-P12 could significantly down-regulate IRF3 phosphorylation in a similar fashion (Figure 5a,d). These results confirmed the inhibitory activity of liposome-loaded HCQ on the TLR4 pathway examined by the reporter cell system.

We next evaluated their anti-inflammatory activities by measuring the pro-inflammatory cytokine production. It was found that both HCQ-L and HCQ-L-P12 treatments strongly reduced the production of the pro-inflammatory cytokines interleukin-6 (IL-6) (Figure 5e), tumor necrosis factor (TNF)-α (Figure 5f), and monocyte chemoattractant protein (MCP)-1 (Figure 5g) upon LPS stimulation for 24 h. These results demonstrate that HCQ-L and HCQ-L-P12 were able to potently down-regulate TLR4-triggered inflammatory responses in macrophages.

As HCQ is known for its ability to modulate endosomal pH, we speculated that the observed TLR inhibitory activity of HCQ-L and HCQ-L-P12 was through attenuating the endosomal acidification. The changes in endosomal pH were examined using two pH-sensitive fluorescent probes: pHrodo red-dextran and fluorescein-dextran. The increase in the ratio of fluorescein to pHrodo red intensities indicates an elevation of endosomal pH. It was clearly seen from the confocal images that the red color (pHrodo red) became dimmer in HCQ-L- and HCQ-L-P12-treated groups than that in the untreated group (Figure 5h), and the ratio of green to red fluorescence in the cells increased significantly (Figure 5i). The same trend was observed in the positive control chloroquine (CQ)-treated cells, which is known for blocking endosomal acidification. These results suggest that HCQ-L and HCQ-L-P12 inhibited intracellular TLR signaling primarily through modulating endosomal pH.

### 3.5. HCQ-L and HCQ-L-P12 Alleviated Lung Inflammation in the LPS-Induced ALI Mouse Model

**Figure 6:**
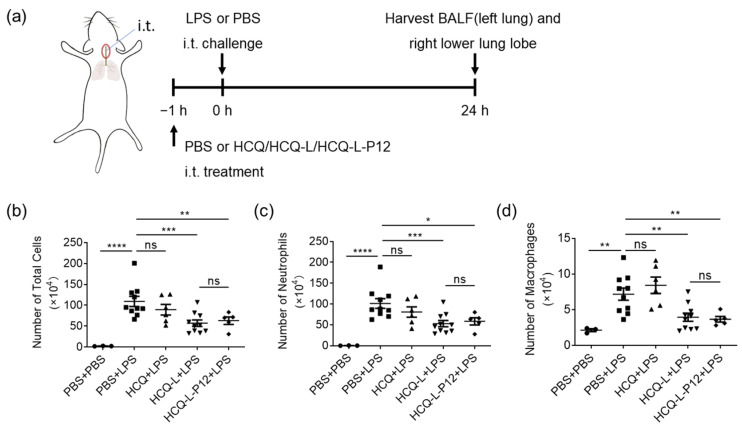
The effects of HCQ-L and HCQ-L-P12 on inflammatory cell infiltration in the BALF of LPS-induced ALI mice. (**a**) The scheme showing the LPS-induced ALI mouse model; the HCQ-L and HCQ-L-P12 treatments were given intratracheally 1 h before LPS challenge (10 mg/kg), and the BALF collected from the left lung was analyzed 24 h later. (**b**–**d**) The analysis of the number of total cells (**b**), neutrophils (**c**), and macrophages (**d**) in the BALF. HCQ/HCQ-L/HCQ-L-P12 = 235.8 μM (HCQ concentration), 50 μL/mouse. *N* = 3 (for the PBS+PBS control group), 10 (for PBS+LPS group), 6 (for HCQ+LPS group), 10 (for HCQ-L+LPS group), 5 (for HCQ-L-P12+LPS group). ns: not significant, * *p* < 0.05, ** *p* < 0.01, *** *p* < 0.001, **** *p* < 0.0001.

**Figure 7:**
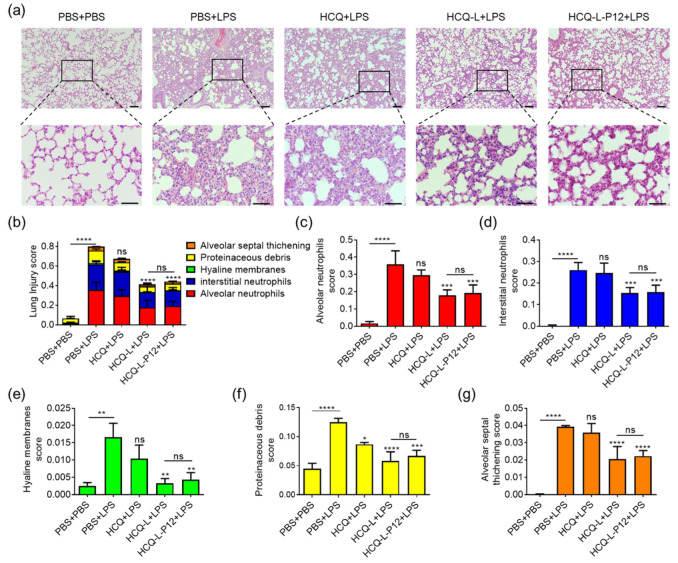
The effects of HCQ-L and HCQ-L-P12 on the lung inflammation and injury of LPS-induced ALI mice. (**a**) The histological images of lung sections stained with H&E; scale bar = 100 μm. (**b**–**g**) The pathological scoring to evaluate the overall lung injury (**b**) based on the 5 pathophysiological characteristics: the alveolar neutrophils (**c**), interstitial neutrophils (**d**), hyaline membranes (**e**), proteinaceous debris (**f**), and alveolar septal thickening (**g**). LPS = 10 mg/kg, HCQ/HCQ-L/HCQ-L-P12 = 235.8 μM (HCQ concentration), 50 μL/mouse; *N* = 5, ns: not significant, * *p* < 0.05, ** *p* < 0.01, *** *p* < 0.001, **** *p* < 0.0001 vs. LPS group unless otherwise specified.

Excessive TLR activation in macrophages plays a key role in the pathogenesis of ALI/ARDS [25]. As HCQ-L and HCQ-L-P12 exhibited potent TLR inhibitory activity in macrophages, it was expected that they may have protective effects on LPS-induced lung injury in vivo. A classical LPS-induced ALI mouse model was employed, where the treatments of molecular HCQ, HCQ-L, and HCQ-L-P12 were given intratracheally 1 h prior to the LPS challenge through the same route; the lung inflammation and injury were assessed at 24 h post LPS challenge (Figure 6a). Upon the LPS challenge, the mouse lungs became inflamed with the elevation of infiltrated immune cells in the bronchoalveolar lavage fluids (BALFs), including the total cells (Figure 6b), neutrophils (Figure 6c), and macrophages (Figure 6d). This increased immune cell infiltration was significantly reduced by both HCQ-L and HCQ-L-P12 treatments but not by the molecular HCQ at the same dose (Figure 6b–d). The histopathological analysis on the mouse lung sections also presented perivascular and peribronchiolar inflammatory cell infiltration upon LPS challenge (Figure 7a), which was reversed by HCQ-L and HCQ-L-P12 but not by HCQ. The lung inflammation and injury were further analyzed by evaluating the lung injury scores (Figure 7b) based on the five features observed in the histological images: alveolar neutrophils (Figure 7c), interstitial neutrophils (Figure 7d), hyaline membranes (Figure 7e), proteinaceous debris (Figure 7f), and alveolar septal thickening (Figure 7g). Again, these elevated lung injury scores by LPS challenge were significantly decreased upon treatment with HCQ-L and HCQ-L-P12; the molecular HCQ showed minute effects on these scores except the proteinaceous debris (Figure 7b–g). These in vivo results indicate that the liposomal HCQ possessed more potent anti-inflammatory activity than the molecular HCQ for controlling acute lung inflammation.

## 4. Discussion

In this study, we engineered a macrophage-targeted, peptide-functionalized liposomal nanoformulation for hydroxychloroquine (HCQ-L-P12) that effectively alleviates acute lung injury by specifically dampening endosomal TLR signaling through the manipulation of endosomal pH. We demonstrated that the simple, effective repurposing of HCQ to treat ALI by addressing its critical limitations through nanotechnology resulted in a nanotherapeutic agent with enhanced specificity, potency, and remarkable formulation stability. This novel nanodrug to manipulate the endosomal pH of macrophages represents a promising, highly effective therapeutic strategy to manage the uncontrolled inflammation in ALI and other inflammatory diseases.

### 4.1. The Important Role of Endosomal TLR Hyperactivation in ALI

The pathogenesis of ALI/ARDS is characterized by an uncontrolled inflammatory cascade, largely driven by resident and infiltrating macrophages [26]. A critical trigger of this cascade is the hyperactivation of endosomal TLRs (e.g., TLR3, TLR4, TLR7/8, TLR9), which recognize a variety of pathogen-derived and endogenous nucleic acids released upon cellular damage [27,28,29]. While essential for host defense, this signaling pathway, when dysregulated, leads to a detrimental “cytokine storm” that destroys the alveolar–capillary barrier [30]. The absence of specific inhibitors for these intracellular receptors in clinics represents a significant unmet medical need. To directly address this gap, we fabricated a physiologically stable nanodevice, HCQ-L-P12, and demonstrated that it could potently suppress signaling of multiple endosomal TLRs (TLR3, 4, and 7/8) and the subsequent pro-inflammatory cytokine production (Figure 4 and Figure 5). The efficacy of our nanoformulation in reducing lung inflammation and injury in the ALI mouse model provides compelling pharmacological evidence that macrophage-targeted intervention against the endosomal TLR axis is a viable strategy to reverse the detrimental immune responses in ALI (Figure 6 and Figure 7).

### 4.2. The Mechanism of Action for the Nanoformulation-Enhanced Therapeutic Efficacy of HCQ in ALI

The activation of endosomal TLRs is exquisitely dependent on the progressively acidic pH of the endolysosomal compartment. This acidic environment is indispensable for the proteolytic cleavage of TLRs like TLR3/7/8/9, the conformational changes required for ligand recognition and receptor dimerization, and the recruitment of downstream adaptor proteins [31]. While weak bases like HCQ have long been known to modulate endolysosomal pH and broadly inhibit immune activation, their use has been limited by a lack of specificity and potential side effects caused by high systemic doses [32]. Without an effective nanocarrier, HCQ mainly exerts its effect by passively diffusing into acidic compartments to sequester protons, thereby attenuating the endosomal pH.

Our research provides a significant advance by demonstrating that nanoformulations of HCQ can leverage this mechanism for enhanced therapeutic outcome. We found that the nanoform of HCQ (HCQ-L) as well as the Pep12-modifiied HCQ-L-P12 significantly enhanced the inhibitory potency against endosomal TLR4 signaling (Figure 1e,f and Figure 4c,d), and both of them effectively elevated the endosomal pH (Figure 5h,i). This intriguing observation suggests that the nanoformulation does not simply serve as a passive carrier but actively improves the distribution and effectiveness of HCQ. These liposomal nanosystems may promote the cellular uptake and potentially provide a sustained release of HCQ within the target cells, leading to a more profound and prolonged alteration of the endosomal pH. This, in turn, results in a more efficient and specific disruption of the endosomal TLR signaling. The fact that Pep12 modification concurrently confers remarkable long-term stability of the formulation and augments its inhibitory profile (for TLR7/8) underscores the potential of peptide-functionalized nanocarriers for the fine-tuning of intracellular signaling pathways.

### 4.3. Broad Therapeutic Prospects of the Peptide-Modified Liposomal Nanosystem for Endosomal pH-Modulating Immunotherapy

The translation of endosomal TLR inhibitors is hindered by the challenge of achieving targeted delivery to the relevant immune cells while avoiding systemic side effects. Our Pep12-modified liposomal nanosystem directly overcomes this hurdle. By formulating HCQ into a liposome, we improved its therapeutic index and reduced the cytotoxicity. The subsequent Pep12 functionalization was a critical breakthrough, conferring superior aqueous stability for at least two years—a vital attribute for clinical translation and commercialization. The success of the intratracheally administered HCQ-L-P12 in the LPS-induced ALI model underscores the power of local delivery to maximize lung bioavailability and minimize off-target effects. Looking forward, the implications of this platform technology extend far beyond ALI. It is known that many chronic inflammatory and autoimmune diseases, including rheumatoid arthritis, systemic lupus erythematosus, and atherosclerosis, are driven by aberrant endosomal TLR activation (e.g., by self-nucleic acids) [33,34,35]. Our stable, targeted liposomal system provides a versatile option. By modifying the surface ligand, this peptide-modified nanosystem can be adapted to target macrophages or other antigen-presenting cells in different tissues, offering a promising new modality for treating these inflammatory conditions by resetting their dysregulated endosomal signaling landscape.

## 5. Conclusions

We developed a lipid-based nanocarrier for HCQ (HCQ-L) to reduce the cytotoxicity of the molecular HCQ and improve its macrophage-targeting ability for the control of excessive acute lung inflammation. It was found that HCQ-L was less cytotoxic and more potent in regulating endosomal TLR signaling-mediated inflammatory responses than the molecular HCQ in THP-1 cell-derived macrophages and in the LPS-induced ALI mouse model. The additional peptide modification on HCQ-L (as HCQ-L-P12) greatly improved the stability of HCQ-L at the physiological condition for at least two years; HCQ-L-P12 exhibited similar quality in endosomal TLR inhibition in vitro and anti-inflammation in mice in comparison with the unmodified HCQ-L. This research not only provides a potent, stable, and macrophage-targeting nanotherapy for ALI, but also deepens our mechanistic understanding of how nanocarriers can be engineered to manipulate intracellular signaling hubs in human diseases.

## Data Availability

The original contributions presented in the study are included in the article, further inquiries can be directed to the corresponding author.
